# The Influence and Mechanisms of Natural Plant Polysaccharides on Intestinal Microbiota-Mediated Metabolic Disorders

**DOI:** 10.3390/foods13233882

**Published:** 2024-11-30

**Authors:** Yong Chen, Hui Li, Furao Lai, Tian Min, Hui Wu, Qiping Zhan

**Affiliations:** 1 College of Food Science and Engineering, South China University of Technology, Guangzhou 510640, China; chenyong@gxnun.edu.cn (Y.C.);; 2 College of Chemical and Biological Engineering, Guangxi Minzu Normal University, Chongzuo 532200, China; 3 Culinary Institute, Shunde Polytechnic, Foshan 528000, China; 4 College of Food Science and Technology, Nanjing Agricultural University, Nanjing 210095, China

**Keywords:** natural plant polysaccharides, gut microbiota, short-chain fatty acids

## Abstract

Natural plant polysaccharides are renowned for their broad spectrum of biological activities, making them invaluable in both the pharmaceutical and food industries. Their safety, characterized by low toxicity and minimal side effects, coupled with their potential therapeutic properties, positions them as crucial elements in health-related applications. The functional effectiveness of these polysaccharides is deeply connected to their structural attributes, including molecular weight, monosaccharide components, and types of glycosidic bonds. These structural elements influence how polysaccharides interact with the gut microbiota, potentially alleviating various metabolic and inflammatory disorders such as inflammatory bowel disease, diabetes, liver-associated pathologies, obesity, and kidney diseases. The polysaccharides operate through a range of biological mechanisms. They enhance the formation of short-chain fatty acids, which are pivotal in keeping intestinal health and metabolic balance. Additionally, they strengthen the intestinal mucosal barrier, crucial for deterring the ingress of pathogens and toxins into the host system. By modulating the immune responses within the gut, they help in managing immune-mediated disorders, and their role in activating specific cellular signaling pathways further underscores their therapeutic potential. The review delves into the intricate structure–activity relationships of various natural polysaccharides and their interactions with the intestinal flora. By understanding these relationships, the scientific community can develop targeted strategies for the use of polysaccharides in therapeutics, potentially leading to innovative treatments for a range of diseases. Furthermore, the insights gained can drive the advancement of research in natural polysaccharide applications, providing direction for novel dietary supplements and functional foods designed to support gut health and overall well-being.

## 1. Introduction

Polysaccharides are an abundant class of natural macromolecules in nature, widely derived from animals, plants, algae, and microorganisms. Natural polysaccharides are formed by the condensation of more than a dozen monosaccharide molecules through dehydration reactions and are one of the basic substances that make up living organisms. Natural plant polysaccharides are macromolecular polymers derived from plant cell walls or intercellular matrices [1]. These primarily include starch, cellulose, hemicellulose, and pectin. Starch and cellulose, solely composed of glucose molecules, are classified as homopolysaccharides; whereas hemicellulose and pectin, composed of two or more types of monosaccharides, are categorized as heteropolysaccharides. As a form of dietary fiber, plant polysaccharides play a crucial role in promoting intestinal motility and maintaining gut health. Due to the human body’s lack of specific degradative enzymes, heteropolysaccharides cannot be directly digested into monosaccharides or oligosaccharides by humans. However, they can be metabolized by intestinal microbes such as Firmicutes and Bacteroidetes through glycolysis [2]. Natural polysaccharides are formed by the condensation of more than a dozen monosaccharide molecules through dehydration reactions and are one of the basic substances that make up living organisms. The broad spectrum of health-promoting activities associated with polysaccharides, including antioxidant [3], anticancer [4,5], immunomodulatory [6], antimutagenic [7], anti-inflammatory [8], antimicrobial [9], anticoagulant [10], hypoglycemic [11], hypolipidemic [12], and hepatoprotective effects [13], has garnered substantial attention in biochemistry and medical research.

As a major source of energy and nutrition for intestinal flora, polysaccharides can significantly regulate the balance of intestinal flora, inhibit the disorder of intestinal flora, and have a positive effect on the intestinal health of the host. Although the body is unable to produce the enzymes needed to directly digest most polysaccharides, the gut microbiome is able to convert these complex carbohydrates into fermentable monosaccharides by encoding enzymes. These simpler monosaccharides are then further metabolized by specific intestinal microbes to form short-chain fatty acids (SCFAs) and a variety of other significant metabolic byproducts [14].

In recent years, more and more attention has been paid to the effects of the interactions between plant polysaccharides and intestinal flora on diseases, especially the relationship between the imbalance of intestinal flora and the occurrence of metabolic diseases. Metabolic syndrome, which includes a spectrum of conditions such as lipid imbalances, hyperglycemia, insulin resistance, and inflammation, is linked to severe health disorders including obesity, diabetes, non-alcoholic fatty liver disease (NAFLD), and osteoarthritis [15,16]. These conditions often exhibit interconnectivity; for example, obesity is a known risk factor for type 2 diabetes (T2DM), and overweight can also contribute to the development of NAFLD [17]. Current research posits that dysbiosis of the gut microbiota is a critical factor in these metabolic disorders, suggesting that its targeted modulation may provide a viable therapeutic strategy. Relevant studies in recent years have shown that intestinal flora imbalance is closely related to the development of metabolic diseases [18,19]. Therefore, regulating the balance of intestinal flora is a new idea to prevent the occurrence of metabolic diseases or slow down their development [20,21]. The stability of intestinal flora is not only crucial to the health of the digestive system but also an important part of the human metabolic system. Metabolic function can be improved by regulating the balance between beneficial intestinal bacteria and potential pathogenic bacteria [20]. At the same time, the bioactive molecules produced by intestinal flora during metabolism can be transported through the intestinal cavity into the metabolic cycle and further affect other metabolic processes as specific ligands [22]. In this process, intestinal integrity plays an important barrier role in blocking harmful metabolites from entering the circulation [23]. This review aims to delve deeply into the structural impacts of polysaccharides on intestinal flora, detailing their influence on various diseases such as inflammatory bowel disease (IBD), diabetes, liver fibrosis, obesity, and kidney disease. By highlighting these relationships, the review seeks to provide valuable insights and reference points for future research and the development of new polysaccharide-based therapeutic approaches.

## 2. Methods of Review

Science Direct, PubMed, Springer, and Web of Science databases were thoroughly searched to find relevant reviews and research reports. Keywords such as “natural plant polysaccharides”, “intestinal flora”, “short-chain fatty acids”, “inflammatory bowel disease”, “diabetes”, ”hepatic fibrosis”, ”obesity”, and “nephropathy” were searched. These articles were thoroughly read to ensure that the selected results address the research topic. The data evaluated included the author, year of publication, objectives, methodology, and findings. Exclusion criteria included literature reviews that do not have full text and literature that does not conform to the core topic. After classification analysis of the retrieved literature and investigation of the studies, 40 studies were selected to be included in this review. The full papers were then reviewed to ensure that they fit the topic of the current investigation. The selected literature was published after 2005.

## 3. Relationship Between Polysaccharides and Intestinal Flora

The human gut has a complex microbial community, containing about 1000 kinds of microorganisms, which transcribe more than 3 million genes and produce thousands of metabolites involved in the normal metabolic process of the human body. The gut microbiota is predominantly composed of four major groups: *Firmicutes*, *Proteobacteria*, *Bacteroidetes*, and *Actinobacteria*, with *Firmicutes* and *Bacteroidetes* forming over 90% of the total gut flora [24]. In a healthy individual, these microbial populations maintain a delicate balance between beneficial and pathogenic bacteria. An imbalance can lead to an overgrowth of pathogenic bacteria, which may contribute to disease development through various direct and indirect mechanisms [25].

**Figure 1:**
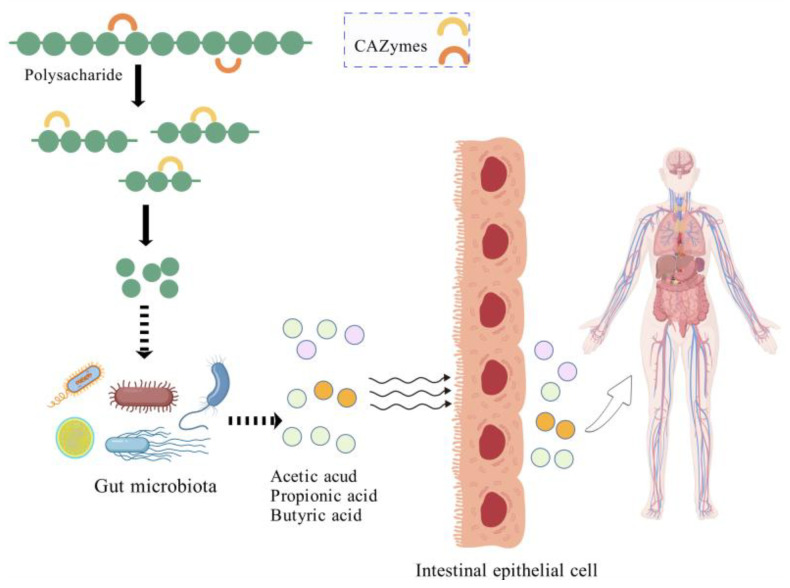
Schematic diagram of CAZymes degrading polysaccharides. (Created with BioGDP.com). URL (accessed on 12 November 2024).

**Figure 2:**
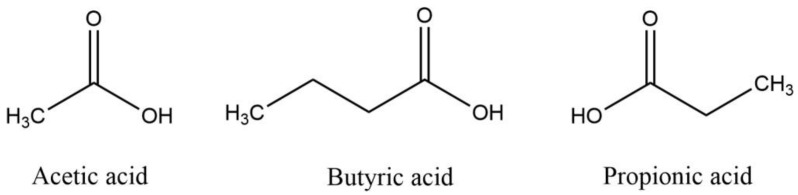
Structure of acetic acid, butyric acid, and propionic acid produced by gut microbial metabolism.

Polysaccharides cannot be directly digested and absorbed by the human digestive system due to their complex structure and high relative molecular weight. Instead, they are metabolized by the gut microbiota through a stepwise degradation process by carbohydrate-active enzymes (CAZymes). CAZymes encoded by gut microbiota include glycoside hydrolases (GHs), glycosyltransferases (GTs), polysaccharide lyases (PLs), carbohydrate esterases (CEs), and carbohydrate binding modules (CBMs). This process breaks polysaccharides down into oligosaccharides and monosaccharides, which are more readily absorbed by the body. Figure 1 shows the degradation of polysaccharides by CAZymes. Concurrently, this fermentation process produces vital metabolic byproducts such as SCFAs, including acetic acid, butyric acid, and propionic acid, which are illustrated in Figure 2 [26]. SCFAs play crucial roles in energy utilization, signal transduction, and metabolic regulation. Moreover, the intake of polysaccharides can modify the gut environment by altering factors like pH and oxygen levels, thus impacting the viability and proliferation of intestinal microflora. Intestinal flora disorders are associated with various diseases, and polysaccharides, as prebiotics, can help prevent diseases or slow down the progression of diseases by regulating the balance of intestinal microflora. Table 1 provides a summary of specific gut flora involved in the production of acetic acid, butyric acid, and propionic acid.

## 4. Physicochemical Properties of Natural Plant Polysaccharides

### 4.1. Molecular Weight

Relative molecular weight is one of the important indicators for the structural characterization of polysaccharides, and the relative molecular weight of plant-derived polysaccharide macromolecules can widely vary, usually ranging from 10 to 10^4^ kDa. The variation not only affects their physical properties but also determines their interaction with biological systems and their ability to elicit specific physiological responses. The comparison of polysaccharides with different molecular weights is shown in Table 2. For instance, in the study of *Rosa setate x Rosa rugosa*, two distinct polysaccharide fractions, WSRP-2a and WSRP-2b, were isolated through anion exchange and gel filtration chromatography. The molecular weights of these fractions were determined to be 56.8 kDa and 23.9 kDa, respectively. Interestingly, the fraction with the lower molecular weight, WSRP-2b, demonstrated a more pronounced inhibitory activity against α-amylase and α-glucosidase enzymes, suggesting that lower-molecular-weight polysaccharides might be more effective in interacting with these biological targets [31]. The conclusion was further supported by the study of leaf polysaccharides from *Dendrobium officinale*, in which two purified polysaccharides with different molecular weights, LDOP-A and LDOP-B, were analyzed for their hypoglycemic effects on diabetes. The results showed that LDOP-A, which had a lower molecular weight, was more effective in lowering blood glucose. Meanwhile, LDOP-A increased butyrate levels, reduced the ratio of *Firmicutes* to *Bacteroidetes*, and increased the abundance of beneficial gut bacteria such as *Lactobacillus*, *Bifidobacterium*, and *Akkermansia* [32]. This underscored the significant impact of molecular weight on the polysaccharide’s ability to modulate the gut microbiome and metabolic health. In another instance, *Astragali Radix* polysaccharides were separated into three distinct molecular weight fragments via ultrafiltration: greater than 2000 kDa (APS-I), around 10 kDa (APS-II), and approximately 300 kDa (APS-III). Each of these fragments exhibited unique bioactivities that correlated with their monosaccharide residue attachment sites, with APS-II demonstrating the strongest immunoenhancement activity [33]. This highlighted the delicate relationship between the relative molecular mass and biological activity of polysaccharides, suggesting some correlation between the two. Polysaccharides with large molecular weight have difficulty to directly cross the cell membrane and enter the cell to exert their biological activities, while polysaccharides with low molecular weight can better exert their activities by regulating the intestinal flora. Polysaccharides with low molecular weight had a suitable molecular weight range to form polymer structures [34]. However, if the molecular mass was too low, a polymer structure capable of generating activity cannot be formed [35].

### 4.2. Glycosidic Linkage and Monosaccharide Composition

Polysaccharides are complex macromolecules composed of monosaccharides connected by glycosidic bonds. The linkage of glycosidic bonds has an important effect on their structural conformation and biological activity. Common linkage types in plant polysaccharides are (1→3), (1→4), and (1→6). Three distinct polysaccharide fractions were extracted from areca nut seeds. The neutral fraction, ACSP-0, exhibited the highest yield. Its molecular structure was characterized by a main chain consisting of →4)-α-D-Glcp- (1→, α-D-Glcp-(1→, →3,4)-α-D-Glcp-(1→ and →4)-β-D-Glcp, with side chains featuring α-D-Glcp-(1→ linkages. The in vitro fecal fermentation of ACSP-0 demonstrated an increase in the levels of SCFAs, fostering the growth of beneficial bacterial strains such as *norank_f_norank_o_Clostridium UCG-014*, highlighting its prebiotic potential [36]. Another potent molecule, PCPs-I, sourced from *Polygonum cuspidatum*, has been identified to alleviate glycemic irregularity and modulate gut microbiota in diabetic mice, with potential therapeutic effects on T2DM [37]. It consisted of glycosidic linkages including →4)-α-D-Glcp-(1→ and →6)-α-D-Glcp-(1→, with minor components of →4,6)-α-D-Glcp-(1→ forming its main chain. The branched structures contained β-D-Glcp-(1→ linked to the sugar residue (SR) at the O-6 position →4,6)-α-D-Glcp-(1→. In addition, an acidic polysaccharide known as FVP-7A was isolated from *Fucus vesiculosus*, primarily comprising →4)-β-D-Manp-(1→, →3)-α-L-Fucp-(1→, α-D-Manp-(1→, →3)-β-D-Manp-(1→ and →4,6)-α-D-Manp-(1→ bonds. FVP-7A notably enhanced the production of SCFAs, especially acetic acid, butyric acid, and valeric acid. It significantly elevated the relative abundance of SCFA-producing bacteria such as *Bacteroides*, *Lachnospira*, *Faecalibacterium*, *Ruminococcus*, *Oscillospira*, and *Dialister* while concurrently suppressing the proliferation of pathogenic Shigella [38]. Taken together, other studies have shown that diglucose α(1–1) fermentation results in higher butyrate levels and a lower proportion of acetate compared with other α-bonded diglucoses. Moreover, diglucose β(1–4) not only increased butyrate production but also enhanced the levels of propionate and butyrate, demonstrating the nuanced impact of specific glycosidic linkages on the metabolic processes within the gut [39].

Plant polysaccharides vary in monosaccharide composition and ratio, and their biological activities are affected by the monosaccharide composition, the molar ratio of monosaccharide, and the uronic acid content. For instance Pleurotus eryngii polysaccharide has a monosaccharide composition of glucose, mannose, galactose, glucuronic acid, and caramel. Pleurotus erinarum polysaccharide has been shown to alleviate liver inflammation, enhance gut barrier function, and normalize the gut microbiota of ducks exposed to aflatoxin b1, a common contaminant in poultry feed [40]. The monosaccharide composition and the monosaccharide molar ratio of guava polysaccharide are given as follows: arabinose (23.42%), galactose (22.42%), galacturonic acid (22.51%), glucose (9.79%), xylose (9.61%), rhamnose (4.40%), glucuronic acid (3.94%), mannose (2.10%), and caramelose (1.81%). It can enhance the bioconversion of arachidonic acid and its metabolites in the liver by reducing harmful bacteria (Desulfovibrionaceae family) and increasing SCFA-producing bacteria (*Bifidobacterium*, *Bacteroides* and *Akkermansia*) to reduce blood glucose [41]. The research results of Auricularia auricularia polysaccharide (AAP) rich in mannose showed that it can significantly reduce the production of AAP by intestinal microbiota in mice, thereby affecting energy intake, and the research results were helpful for weight management strategies for obesity [42]. Yu et al. [43] believed that the order of utilization of the monosaccharide components of beet pulp polysaccharide in intestinal flora was arabinose, glucose, caramel, and galacturonic acid. The content of arabinose and galactose was positively correlated with the yield of butyric acid, while the content of galacturonic acid was negatively correlated with the yield of butyric acid. Mueller et al. [44] confirmed that one of the reasons for the better prebiotic activity of the neutral polysaccharide fraction isolated from the *Hyptis suaveolens* seed was the high galactose content in its monosaccharide composition.

Neutral monosaccharides are typically utilized first, providing immediate energy sources, while acidic monosaccharides are metabolized slower, potentially impacting longer-term gut health and stability. This sequence affects the production of key metabolites like SCFAs, which are vital for maintaining gut health and systemic immunity. For example, the neutral sugar component of pistachio shell, which is composed of monosaccharides such as glucose, xylose, fructose, and arabinose, significantly increased the content of acetic acid, propionic acid, and butyric acid produced by *Lactobacillus plantarum* PTCC 1896 and *Lactobacillus rhamnosus* GG [45].

Wu et al. [46] studied the structural differences between homogenized honey-processed *Astragalus* polysaccharides (HAPS3a) and *Astragalus* polysaccharides (APS3a) and their effects on mice with colitis. The molar ratio of galacturonic acid in HAPS3a (33.24%) was lower than that in APS3a (49.55%) because the aldehyde residues 1,4-β-GalpA and 1, 6-α-Glcpa in the APS3a backbone were converted to the corresponding neutral residues in HAPS3a after honey processing. However, HAPS3a with low uronic acid content was superior to APS3a in biological activities such as protecting the intestinal mucosa, affecting the expression of cytokines, and regulating the balance of intestinal flora. Larsen et al. [47] found that high uronic acid content in polysaccharides can significantly reduce the relative abundance of intestinal flora and the availability of pectin. Although some literatures showed that high uronic acid content can inhibit the effectiveness of polysaccharides as prebiotics, some studies have found that polysaccharides with high uronic acid can improve lipid metabolism and intestinal flora imbalance in the obesity of mice fed with high fat diet, indicating that the specific role of uronic acid needs to be considered when designing polysaccharide-based health interventions [25]. The above literature suggested the importance of specific monosaccharide composition in the promotion of gut microbiota balance by polysaccharides and its potential application in therapeutic interventions for metabolic diseases. Further studies on the relationship between the monosaccharide composition, the molar ratio of monosaccharide, and the uronic acid content and biological activities of plant polysaccharides will undoubtedly facilitate more targeted and effective use of polysaccharides in intestinal health and metabolic diseases.

As shown in Table 3, it can be observed that glucose is the predominant constituent of the plant polysaccharides. Due to the types in glycosidic bonds (α/β-glycosidic bonds), glucans are mainly classified into two major types: α-glucans and β-glucans. The specific binding mode of glycosidic bonds can influence the spatial structure and biological activity of glucans. For example, alpha-glucans have probiotic colonization, antioxidant, and immune-activating activities [48,49]. β-Glucans are used in tumor therapy, improving hyperglycemia, enhancing immune response, etc. [50,51].

In addition to glucose, polysaccharides also contain mannose, fucoidan, galactose, etc. These sugars also have very excellent physiological activities (such as being an antioxidant and performing immune regulation). At the same time, together with glucose, they are used as the basic reaction material and are linked to proteins by enzyme reactions to form glycoproteins [52]. N- and O-glycans are the two main glycation classes on eukaryotic proteins. N-glycans usually have a core five-sugar structure with peripheral sugar chains of similar structure [53]. The most common form of this oligosaccharide is hypermannan, such as Man and GlcNAc [54]. N-glycans play important roles in protein stability, folding, serum half-life regulation, and transport to cell subcompartments and specific tissues [55]. O-glycans are oligosaccharides attached to serine, threonine residues, and other amino acid residues in the side chain of protein peptide hydroxyl groups. O-glycans play an important role in regulating protein function, stability, and interaction with other molecules [56].

## 5. Therapeutic and Preventive Effects of Polysaccharides on Various Diseases Mediated via Modulating Intestinal Microbiota

The disruption of the intestinal flora balance can lead to the occurrence of specific metabolic diseases, such as IBD, diabetes, hepatic fibrosis, and so on. As prebiotics, polysaccharides contribute to the improvement of physiological states by regulating the composition and function of the gut microbiota. This section reviews the roles of polysaccharides in gut microbiome-mediated diseases.

### 5.1. Inflammatory Bowel Disease

IBD, encompassing conditions such as Crohn’s disease (CD) and ulcerative colitis (UC), is characterized by chronic intestinal inflammation, primarily mediated by immune system dysregulation. The exact etiology of IBD remains elusive, but the therapeutic potential of polysaccharides in this context is gaining recognition. Polysaccharides with noted anti-inflammatory and immunomodulatory properties have shown significant therapeutic effects on IBD. Table 4 reviews the effect of polysaccharides on gut-microbe-mediated IBD.

*Crataegus pinnatifida* polysaccharide (HAW1-2) has demonstrated the ability to reverse colon pathology; suppress the production of key inflammatory cytokines such as IL-1β, IL-6, and TNF-α; and inhibit the activation pathways of IKKα/β, IκBα, and NF-κB. This polysaccharide also modified the gut microbiota by enriching beneficial *bacteroides* species, including *Aliistipes* and *Odoribacter*, and promoted the production of SCFAs, which were essential for relieving colitis symptoms [57]. *Sagittaria sagittifolia* L. polysaccharides (PSSP-1) actively upregulated the expression of intestinal tight junction proteins, such as claudin-1, occludin, and ZO-1, crucial for maintaining the integrity of the mucosal barrier. These polysaccharides promoted the increase in the relative abundance of beneficial gut bacteria such as *Lactobacillus* and *Candidatus_Saccharimonas* and the increase in SCFA levels, while reducing the relative abundance of *Bacteroidetes* and *Verrucomicrobiota*. In addition, PSSP-1 can inhibit the inflammatory signaling pathways MAPK and NF-κB, thereby contributing to the alleviation of colitis [58]. Bergamota polysaccharide (BP) promoted the metabolic balance of intestinal flora by promoting the growth of beneficial bacteria such as *Bifidobacteria*, *Butyrivibrio*, and *Blautia*. In addition, it increased the expression of tight junction proteins and mucin (MUC2), which strengthened the intestinal barrier and diminished inflammatory responses [59]. Polysaccharides from the aboveground part of Angelica sinensis (ASP-Ag-AP) exhibited protective effects against dextran sodium sulfate (DSS)-induced colitis by inhibiting the TLR4/MyD88/NF-κB signaling pathway and restoring serum levels of the metabolite 5-methyl-dl-tryptophan (5-MT), thus reducing colonic inflammation and enhancing barrier function while modulating the gut microbiota [60]. Ginger polysaccharide (GP) alleviated DSS-induced UC by suppressing pro-inflammatory cytokines such as TNF-α, IL-6, IL-1β, IL-17A, and IFN-γ, and promoting the expression of occludin-1 and ZO-1. Moreover, GP normalized the *Firmicutes/Bacteroidetes* ratio, reduced the populations of *Bacteroides* and *Proteobacteria*, and increased the abundance of *Lactobacillus* and *Verrucomicrobiota*, thereby restoring intestinal microbial balance [61]. Selenium tea polysaccharide (ASeTP) had strong anti-inflammatory and antioxidant properties, which can significantly reduce the levels of pro-inflammatory cytokines and improve the enrichment level of beneficial bacteria. ASeTP also restored the equilibrium of gut microbiota, alleviated symptoms such as weight loss and colon shortening, and elevated disease activity in DSS-induced colitis mice [62]. Fuzhuan Brick Tea Polysaccharide (FBTP) corrected intestinal flora dysbiosis associated with UC, promoted the proliferation of beneficial bacteria like *Lactobacillus* and *Akkermansia*, and increased SCFA levels. FBTP also affected tryptophan metabolism and increased the fecal levels of indole-3-acetaldehyde and indole-3-acetic acid. In addition, FBTP can significantly upregulate the expression of AhR and IL-22 in the colon and enhance the expression of tight junction proteins such as ZO-1 and occludin [63].

**Figure 3:**
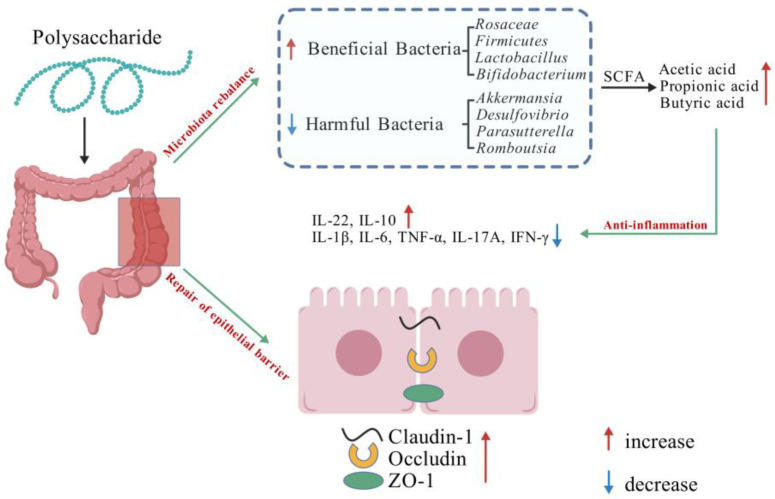
The mechanisms of polysaccharides improving IBD (Created with BioGDP.com). URL (accessed on 30 August 2024).

Collectively, these studies underscored the ability of polysaccharides to enrich beneficial gut bacteria like *Firmicutes*, *Bifidobacterium*, *Lactobacillus*, and *Rosaceae*; diminished harmful bacteria; and maintained intestinal homeostasis (Figure 3). Polysaccharides have been shown to be effective in relieving IBD symptoms by enhancing SCFA production, inhibiting inflammatory mediators, and improving intestinal integrity, thus supporting their potential therapeutic application.

**Table 4:** Effects of polysaccharides on gut-microbe-mediated IBD.

Name	Source	Molecular Weight (kDa)	Monosaccharide Composition	Glycosidic Bond	Gut Microbiota Regulation	References
HAW1– 2	*Crataegus pinnatifida*	8.94	Ara; Gal; Glu	→4)-β-D-Glc*p*-(1→, →4)-β- D-Gal*p*-(1→, α-L-Ara*f*-(1→, →5)-α-L-Ara*f*-(1→, β-D- Glc*p*-(1→ and →4,6)-β-D- Glc*p*-(1→	*Alistipes* and *Odoribacter* ↑	[57,64]
PSSP-1	*Sagittaria sagittifolia* L.	65.79	Glu; Ara; Man; Gal	Ara*f*-(1→, Glc*p*-(1→, Gal*p*- (1→, →2)-Glc*p*-(1→, →6)- Glc*p*-(1→, →4)-Glc*p*-(1→, →2,6)-Man*p*-(1→, →4,6)- Glc*p*-(1→, and →3,6)-Gal*p*- (1→.	*Lactobacillus*, Nitrosospira, *Dia lister* and *Candidatus_Sacchari monas* ↑ *Bacteroidetes*, *Actinobacteria* and *Verrucomicrobiota* ↓	[58]
BP	Bergamot	335	Glu; Xyl; Man; Ara; Gal; GluA		*Alistipes* and *Bacteroides ↓ Butyrivibrio*, *Bifidobacterium*, *Lachnoclostridium* and *Romboutsia* ↑	[59]
ASP-Ag-AP	*Angelica sinensis* aboveground part	51.5	Man; Rha; GluA; GalA; Glu; Gal; Ara; Fuc; Xyl	1,4-linked GalA*p*, 1,2 and 1,2,4-linked Rha*p* and T-Ara*f*	*Bacteroides*, *Streptococcus*, *Fae calibacterium, Staphylococcus*, *Alistipes* ↑ *Lactobacillus* ↓	[60]
GP	Ginger	747.2	Gal; Ara		*Bacteroides and* *Proteobacteria* ↓	[61]

Note: ↑: increase; ↓: decrease.

### 5.2. Diabetes

Diabetes, an endocrine and metabolic disorder impacting over 400 million individuals globally, is characterized by compromised pancreatic beta-cell functionality, insufficient insulin secretion, or insulin resistance [65,66]. This disease primarily manifests in two forms: type 1 diabetes (T1DM) and T2DM [67]. T1DM is an autoimmune disease that targets pancreatic cells and drastically reduces insulin secretion, leading to elevated blood glucose levels, accounting for approximately 10% of diabetes cases. In contrast, T2DM, accounting for about 90% of diabetes cases, arises from reduced tissue sensitivity to insulin, resulting in insulin resistance, hyperglycemia, and insulin deficiency. T2DM is accompanied by severe health complications such as kidney disease, neuropathy, cardiovascular disease, and retinopathy, making it a major public health concern [35,68]. Polysaccharides have significant adhesion properties and can enhance intestinal permeability and improve the bioavailability of other nutrients and efficacy components, providing a promising way for the improvement of T2DM [69]. Table 5 reviews the effect of polysaccharides on gut-microbe-mediated diabetes.

Polysaccharides extracted from *Polygonum cuspidatum* (PCPs-I) showed the ability to reduce insulin resistance and lower total cholesterol (TC), triglyceride (TG), low-density lipoprotein cholesterol (LDL-C), and free fatty acid (FFA) levels in mice with T2DM. These compounds beneficially altered the gut microbiota by reducing the populations of Corynebacterium and Ligilactobacillus while increasing *Oligella* and *Mammaliicoccus*, thereby addressing both glycemic control and intestinal health disruptions typical in diabetic conditions [37]. *Achyrantha bidentata* polysaccharide (ABP) can effectively repair the intestinal barrier and directly affect the gut microbiota, and increase the abundance of SCFA bacteria such as *Alloprevotella*, *Bacteroides*, *Prevotellaceae_UCG_001*, *Prevotellaceae_NK3B31_group*, and *Akkermansia*. This interaction boosted the hypoglycemic effects of ABP by elevating SCFA levels and activating critical biochemical pathways, including GLP-1/GLP-1R/cAMP/PKA/CREB/INS, showcasing its therapeutic potential [70]. Additionally, the research highlighted the differential impacts of polysaccharides according to their molecular weights. LDOP-A and LDOP-B, two polysaccharides isolated from *Dendrobium officinale* leaves, were evaluated for their effects on hyperglycemia and insulin resistance. LDOP-A, with a lower molecular weight, was particularly effective, enhancing colonic SCFA levels; optimizing the *Firmicutes* to *Bacteroidetes* ratio; and promoting the growth of beneficial gut bacteria such as *Lactobacillus*, *Bifidobacterium*, and *Akkermansia* [32]. Furthermore, the hypoglycemic properties of ultra-filtered polysaccharides from *Berberis dasystachya* (UBDP) have been documented. UBDP administration improved glucose tolerance, enhanced organ function, reduced fasting blood glucose levels and lowered the glycosylated hemoglobin index. It also improved pancreatic insulin sensitivity and reduced oxidative stress in diabetic rats, significantly altered gut microbiota composition to favor beneficial Firmicutes, and enhanced SCFA levels [71]. GFP-N, a polysaccharide extracted from maitake mushrooms, effectively lowered fasting blood glucose, improved glucose tolerance, alleviated insulin resistance, and protected against liver and kidney damage. It influenced the IRS1/PI3K and JNK signaling pathways in the liver, contributing to its effects on hepatic insulin resistance. Additionally, it significantly altered gut microbiota composition; increased the abundance of Bacteroidetes and decreased Firmicutes and Proteobacteria; as well as enhanced beneficial species like Akkermansia, Lactobacillus, and Turicibacter [72]. Ultrasonic extracted Codonopsis pilosula crude polysaccharides (CPCPs) can improve abnormal lipid metabolism in T2DM mice by reducing the relative abundance of Enterobacter and increasing the relative abundance of Bacteroides and the contents of TG and LDL-C. CPCPs also can improve the inflammatory response by reducing the expression levels of NF-κB, TNF-α, and IL-6 in T2DM mice and increasing the activities of SOD, GSH-Px, and CAT to reduce oxidative stress in various organs [73].

**Figure 4:**
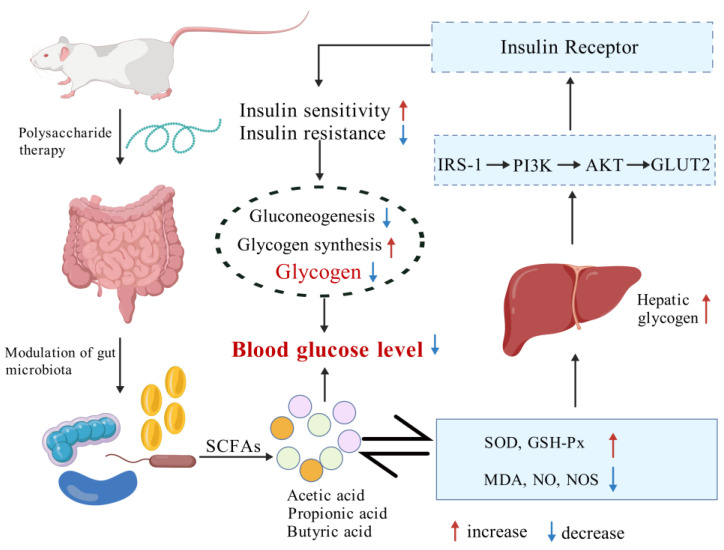
The mechanisms of polysaccharides improving diabetes (Created with BioGDP.com). URL (accessed on 6 October 2024).

The above literature indicated the role of polysaccharides in regulating the balance of gut microbiota, promoting the growth of beneficial bacteria, and enhancing SCFA production. In addition, polysaccharides can reduce intestinal inflammation by inhibiting the expression of inflammatory factors and repairing damaged islet cells (Figure 4). The above results further confirm the potential of polysaccharides as natural products in diabetes applications.

**Table 5:** Effects of polysaccharides on gut-microbe-mediated diabetes.

Name	Source	Molecular Weight (kDa)	Monosaccharide Composition	Glycosidic Bond	Gut Microbiota Regulation	References
PCPs-I	*Polygonum cuspidatum*	68.208	Ara; Glu; Gal; Rha; Fuc	Glc*p*-(1→, →3)-Gal*p*-(1→, →6)-Glc*p*-(1→, →4)-Glc*p*- (1→, →3, 4)-Glc*p*-(1→, →4,6)-Glc*p*-(1 →	*Oligella* and *Mammaliicoccus* ↑ *Corynebacterium* and *Ligilactobacillus* ↓	[37]
ABP	*Achyranthes bidentata*	41.08	Man, Rib, Rha, Glu-UA, Gal- UA, Glu, Gal, Ara, Fuc. Glu, Gal-UA, and Man		*Alloprevotella, Bacteroides, Prevotellaceae_UCG_001, Prevotellaceae_NK3B31_group*, and *Akkermansia* ↑	[70]
LDOP	*Dendrobium officinale* leaf	LDOP-A: 9.91 LDOP-B: 147.45	LDOP-A: Glu; Man; GluA; Gal LDOP-B: Man; Glu; Gal; GluA; Ara		*Bacteroidetes* ↑, *Firmicutes* ↓ LDOP-A: *Clostridiales*, *Akkermansia*, *Bifidobacterium* and *Lactobacillus* ↑	[32]
UBDP	*Berberis dasystachya*	10.2	Man; Ara; Glu; Gal; Xyl; Fru; GulA; GluA; GalA		*Ruminococcaceae NK4A214* group, *Ruminococcus* *2*, *Prevotellaceae NK3B31* group, *Eubacterium* *coprostanoligenes* group, *Rombo utsia*, and *Alloprevotella* ↑	[71]
GFP-N	Maitake mushroom	12600	Ara; Man; Glu	→2,6)-*α*-D-Man*p*-(1 → 4, *α*-L- Ara*f*-C1→, and →3,6)-*β*-D- Glc*p*-(1 →	*Bacteroidetes*, *Akkermansia*, *Lactobacillus*, and *Turicibacter* ↑ *Firmicutes* and *Proteobacteria*↓	[72]
CPCPs	*Codonopsis pilosula*	4.418	Man; Rha; Glc; Gal; Ara		the ratio of *Firmicutes* and *Bacteroidetes, Enterobacter* ↓ *Bacteroides* ↑	[73]

Note: ↑: increase; ↓: decrease.

### 5.3. Hepatic Fibrosis

Hepatic fibrosis is an advanced pathological state induced by various etiological agents such as excessive alcohol consumption, viral and autoimmune hepatitis, NAFLD, and cholestatic liver disease [74]. These causative factors initiate a cascade of damaging processes including inflammation, immune response activation, neurotransmitter release, and hepatic stellate cell (HSC) activation. This results in abnormal connective tissue proliferation and excessive extracellular matrix (ECM) accumulation, leading to the progressive scarring of liver tissue, known as hepatic fibrosis [75]. Complex catabolic and metabolic processes in the gut involve the conversion of endogenous (such as bile acids and amino acids) and exogenous substances (from diet and the environment). These products are transported to the liver via the portal vein, where they are further metabolized and affect liver function [76]. Therefore, liver fibrosis is associated with the homeostasis of the intestinal environment. Table 6 reviews the effect of polysaccharides on gut-microbe-mediated hepatic fibrosis.

In a pivotal study, Li et al. [77] investigated the therapeutic effects of *Radix Puerariae thomsonii* polysaccharide (RPP-2) in a mouse model of diet-induced NAFLD. They discovered that RPP-2, by activating the farnesoid X receptor (FXR) pathway, was effective in regulating the levels of gut microbiota, including *Flintibacter*, *Butyricococcus*, and *Oscillobacteria*, as well as their metabolites such as lipopolysaccharides, bile acids, and SCFAs. Thus, it can significantly alleviate related hyperlipidemia and NAFLD. Similarly, the Gardenia jasminoides Ellis polysaccharide (GPS), when administered in a dose-dependent manner, dramatically improved the hepatic function in models of cholestatic liver damage. This was evidenced by reductions in aminotransferase levels, alleviation of tissue damage, upregulation of critical receptors such as the farnesoid X receptor and pregnane X receptor, and a reduction in bile acid levels. Furthermore, GPS enhanced intestinal barrier integrity, corrected dysbiosis in gut microbiota, and reduced both serum and hepatic levels of lipopolysaccharides. It also effectively downregulated the TLR4/NF-κB signaling pathway, leading to a decrease in the expression of inflammatory genes and a mitigation of hepatic inflammation [78]. Poria cocos (PCP) has shown considerable efficacy in improving symptoms associated with nonalcoholic steatohepatitis, such as histological liver damage, impaired liver function, and increased oxidative stress. PCP achieved these results by modulating the NF-κB/CCL3-CCR1 axis, enriching beneficial probiotics, and reducing the endotoxin load from intestinal bacteria [79]. Garlic polysaccharide has demonstrated significant hepatoprotective effects against alcoholic liver fibrosis in mice by modulating lipid peroxidation and oxidative stress and regulating critical signaling pathways such as TGF-β1, TNF-α, and decorin. This intervention restrained HSC activation and decreased ECM production [80]. Paeoniae alba (RPAPs) not only increased the abundance of beneficial bacteria such as *Lactobacillus*, *Alistipes*, *Bacillus*, and *Rikenellaceae_RC9_gut_group* but also reduced the relative abundance of pathogenic microorganisms, thereby improving lipopolysaccharide-induced intestinal damage [81]. Astragalus polysaccharide (AP) has been effective in reducing the serum levels of AST, ALT, TC, and TG and significantly restored the diversity and community structure of intestinal mucosal bacteria in mice with alcoholic liver disease. Remarkably, AP modulated the *Firmicutes* to *Bacteroidetes* ratio, favoring an increase in beneficial microbial populations and a decrease in pathogenic ones [82]. Fufang Zhenzhu Tiaozhi polysaccharides (FTZPs) and *Codonopsis pilosula* polysaccharides (CPPs) have shown promising results in improving liver lipid metabolism, reducing inflammation and fibrosis, and enhancing the integrity of the intestinal mucosal barrier. In addition, FTZPs have been confirmed to significantly reduce the relative abundance of *Gammaproteobacteria*, *Clostridium*, and *coprococcus* while inducing an increase in the relative abundance of *Dehalobacteraceae* and *Dehalobacterium*, which helped to improve energy metabolism, lipid metabolism, body inflammation, and liver fibrosis in mice with high-fat diet [83]. CPP has been shown to effectively reduce the expression levels of fatty acid uptake genes Cd36 and Fabp1 and adipogenesis-related genes Fasn, Pparγ, Srebp-1c, and Scd1, and restore sterigmatocystin-induced abnormal increases in serum and liver lipid levels in mice. These results indicated that CPP can reduce fatty acid uptake and lipogenesis, and reverse abnormal lipid metabolism [84].

**Figure 5:**
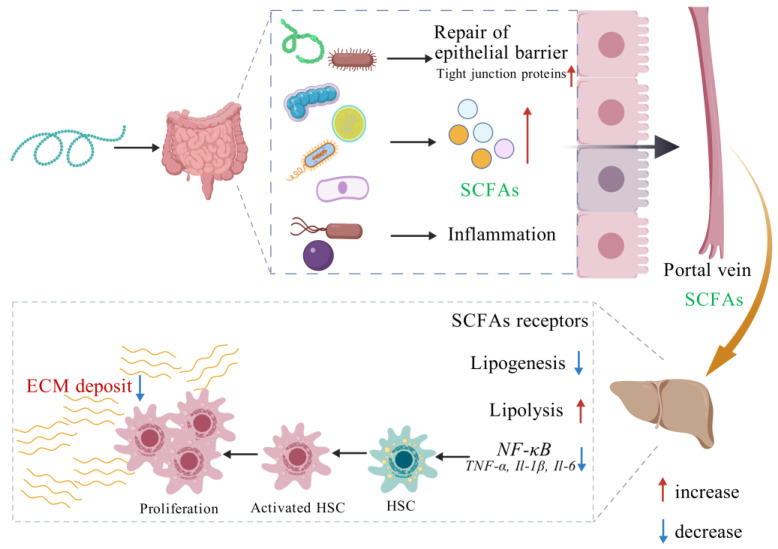
The mechanisms of polysaccharides improving hepatic fibrosis (Created with BioGDP.com). URL (accessed on 6 October 2024).

Taken together, polysaccharides can regulate the balance of intestinal flora and restore the intestinal barrier by increasing the relative abundance of probiotics such as *Bacillus* and *Lactobacillus* and reducing the relative abundance of harmful bacteria. In addition, polysaccharides can also play a role in improving liver fibrosis by reducing inflammation and regulating immune function (Figure 5).

**Table 6:** Effects of polysaccharides on gut-microbe-mediated hepatic fibrosis.

Name	Source	Molecular Weight (kDa)	Monosaccharide Composition	Glycosidic Bond	Gut Microbiota Regulation	References
RPP-2	*Radix Puerariae thomsonii*	10.03	Glu	→3)-Glc*p*-(1→	*Butyricicoccus*, *Flintibacter*, and *Oscillibacter* ↑	[77]
GPS	*Gardenia jasminoides* Ellis	130	Rha; Ara; Xyl; Man; Glu; Gal		*Bacteroidetes* and *Firmicutes* ↑ *Proteobacteria* and *Verrucomicrobia* ↓	[78]
PCP	*Poria cocos*		Gal; Man; D- Glucosamine hydrochloride; Xyl; Ara; Fuc		Faecalibaculum, Escherichia_Shigella, and Oscillospirales ↑	[79]
GP	garlic	10	Fru; Gal; Gal- A		*Lachnospiraceae* and *Lactobacillus* ↑ *Facklamia* and *Firmicutes* ↓	[80]
RPAP S	*Radix paeoniae alba*	100	Glu; Ara; Gal	α-(1 → 4)-Glcp, α-Arap, α- Galp, α-Rhap, β-D-Glcp, α-(1 → 6)-linked Glcp and GalA	*Lactobacillus*, *Alistipes*, *Bacillus*, *Rikenellaceae*_RC9_gut_group ↑ *Klebsiella*, *Helicobacter*, *Enteroc* *occus* ↓	[81,85]
FTZPs	Fufang Zhenzhu Tiaozhi		Ara; Glu; Gal; GalA; Man; Xyl; Fuc; GluA		*Gammaproteobacteria*, *Clostridium*, and *Coprococcus* ↓ *Dehalobacteraceae* and *Dehalobacterium* ↑	[83,86]
CPP	Codonopsis pilosula	133.2	Glu; Gal; Ara	(1→4)-linked Glc*p* (residue A), (1→6)-linked Gal*p* (residue B), (1→2,6)-linked Glc*p* (residue C), (1→5)-linked Ara*f* (residue D), and non-reducing terminal (1→)-linked Glc*p* (residue E)	*Firmicutes* and *Proteobacteria* ↓ *Bacteroidetes*, *Actinobacteria* and *Verrucomicrobiota* ↑	[84,87]

Note: ↑: increase; ↓: decrease.

### 5.4. Obesity

The potential of polysaccharides in the treatment of obesity is an area of intense interest. Obesity is a metabolic symptom caused by excessive accumulation and abnormal distribution of fat in the body, which is often accompanied by weight gain [88]. It is an important risk factor for metabolic syndrome such as type II diabetes, coronary heart disease, hypertension, and chronic renal failure. In recent years, a number of studies have shown that polysaccharides have anti-obesity activity and can regulate lipid metabolism through a variety of mechanisms so as to show significant effects in the treatment of obesity [89].

Specifically, Lycium barbarum polysaccharides can be used as a prebiotic agent to improve obesity by modulating the composition of intestinal flora and the metabolism of short-chain fatty acids [90]. *Auricularia auricula* polysaccharides selectively enhanced *Papillibacter cinnamivorans*, reduced fat depositing, and enhanced glucose tolerance [91]. Metabolites of Clostridium leptum fermenting flaxseed polysaccharide can alleviate obesity in rats [92]. Oolong tea polysaccharides inhibited obesity through effects on the pathways of fatty acid biosynthesis and metabolism [93].

In brief, the possible anti-obesity mechanisms of polysaccharides mainly include inhibition of fat absorption, alteration of intestinal microbiota and its metabolites (such as short-chain fatty acids), and regulation of appetite and satiety [94]. However, future studies are needed to further explore the specific mechanism of action, safety, and long-term effects of polysaccharides in order to promote their widespread use in the field of obesity treatment.

### 5.5. Nephropathy

As for the application of polysaccharide in the treatment of kidney disease, its potential is huge. The relevant research is still in the initial stage, and polysaccharide cannot be directly used as a specific drug in the treatment of kidney disease, but more as an auxiliary treatment. In the treatment of nephropathy, polysaccharides play a role mainly through immune regulation, being an antioxidant and anti-inflammatory, and so on [95].

Astragalus polysaccharide attenuates diabetic nephropathy by reducing apoptosis and enhancing autophagy [96]. Sanziguben polysaccharides inhibit diabetic nephropathy through anti-inflammation [97]. *Lycium barbarum* polysaccharides ameliorate renal injury and inflammatory reaction in alloxan-induced diabetic nephropathy rabbits [98]. Of course, in recent years, intervention targeting intestinal flora may provide new ideas for the treatment of chronic kidney disease. The regulation of intestinal microecological balance through probiotics and prebiotics may help to reduce kidney inflammatory response and damage. Modulating the gut microbiota and inflammation is involved in the positive effect of *Bupleurum* polysaccharides against diabetic nephropathy in mice [99]. Polysaccharides from Sacha Inchi shell reduces renal fibrosis in mice by modulating intestinal microbiota [100]. However, although polysaccharides show some potential in the treatment of kidney disease, the safety and efficacy of polysaccharides in the treatment of kidney disease need to be verified through more clinical trials.

## 6. Conclusions

The gut microbiota is increasingly acknowledged as a critical element in sustaining overall host health, intricately involved in a wide range of metabolic, immune, and cellular processes. Recent advances in research have highlighted the complex interplay between diet, gut microbiota, and health outcomes, positioning the application of natural product active ingredients, especially polysaccharides, as a key focus in contemporary scientific exploration and health interventions.

In this review, we explored the effects of natural plant polysaccharides on the balance of gut microbiota and analyzed their potential in the prevention and improvement of IBD, diabetes, liver fibrosis, obesity, and nephropathy. Each section has contributed to an enriched understanding of how dietary polysaccharides influence gut microbiota composition, enhance intestinal barrier functions, modulate immune responses, and ameliorate disease manifestations. The findings underscore the remarkable potential of polysaccharides to act as prebiotics that promote beneficial microbial growth while inhibiting pathogenic bacteria, thereby supporting the maintenance of intestinal equilibrium. These insights pave the way for promising future research directions, including the development of specific dietary strategies that utilize the prebiotic properties of polysaccharides to combat or improve chronic diseases. Moreover, investigating the molecular interactions between specific polysaccharides and the gut microbiota can reveal new therapeutic targets and mechanisms. In summary, this article not only reaffirms the pivotal role of the gut microbiota in maintaining host health but also highlights the critical role of natural polysaccharides in the future landscape of dietary therapy and microbiota management. As the field of gut microbiota research progresses, it is anticipated that innovative applications for polysaccharides will emerge, expanding their applicability in medical science and nutritional therapy, and offering new horizons for health enhancement and disease prevention.

## Figures and Tables

**Table 1:** Intestinal flora producing acetic acid, propionic acid, and butyric acid.

SCFAs	Producers	References
Acetic acid	*Blautia hydrogenotrophica*, *Faecalibacterium prausnitzii*, *Roseburia* spp., *Eubacterium rectale*, *Eubacterium hallii*, *Anaerostipes hadrus*, *Coprococcus catus*, *Bifidobacterium longum*, *Akkermansia muciniphila*	[27,28]
Butyric acid	*Faecalibacterium prausnitzii*, *Roseburia* spp., *Eubacterium rectale*, *Eubacterium hallii*, *Anaerostipes* spp., *Eubacterium hallii*, *Anaerostipes* spp.	[27,29]
Propionic acid	*Phascolarctobacterium succinatutens*, *Dialister* spp., *Veillonella* spp., *Roseburia inulinivorans*, *Ruminococcus obeum*	[27,30]

**Table 2:** Comparison of molecular weight differences among different polysaccharide components extracted from the same plant.

Name	Source	Molecular Weight (kDa)	References
WSRP-2a	*Rosa setate x Rosa rugosa*	56.8	[31]
WSRP-2b		23.9	
LDOP-A	*Dendrobium officinale*	9.91	[32]
LDOP-B		147.45	
APS-I	*Astragali Radix*	2000	[33]
APS-II		10	
APS-III		300	

**Table 3:** Comparison of different glycosidic linkage and monosaccharide composition of polysaccharides.

Name	Source	Presence Site	Average Molecular Weight (kDa)	Monosaccharide Composition	Glycosidic Bond	References
ACSP-0	Areca nut (*Areca catechu* L.)	Seed	4.145	Glc	→4)α-D-Glcp-(1→, α-D-Glcp-(1→, →3,4)α-D-Glcp-(1→ and →4)β-D-Glcp andα-D-Glcp-(1 →.	[36]
PCPs-I	*Polygonum cuspidatum*	Complete plant	68.208	Glc	d →4)-α-D-Glcp-(1→,→6)-α-D-Glcp-(1→ and →4,6)-α-D-Glcp-(1→,β-D-Glcp-(1→ and→4,6)-α-D-Glcp-(1→.	[37]
FVP-7 A	*Fucus vesiculosus*	Complete plant	30.94	Man, Fuc	→4)-β-D-Manp-(1→, →3)-α-L-Fucp-(1→, α-D-Manp-(1→, →3)-β-D-Manp-(1 → and →4,6)-α-D-Manp-(1→.	[38]
PE polysaccharides	*Pleurotus eryngii*	Complete plant	-	Glu, Man, Gal, GluA, Fuc	-	[40]
GP	*Psidium guajava ‘Pearl’*	Complete plant	1386.38, 752.38	Ara, Gal, GalA, Glu, Xyl, Rha, GluA, Man, Fuc	-	[41]
AAP	*Auricularia auricula*	Complete plant	-	Man, GluA, Xyl	-	[42]
W, CA, SC, SH01, SH05	Sugar beet	Pulp	195, 203, 455, 969, 864	Ara, Gal, GalA, Rha	-	[43]
TPS	Lamiaceae (*Hyptis suaveolens*)	Seed mucilage	13.4	Gal, Glu, Man, Fuc, Xyl, 4-O-methylglucuronic acid	-	[44]
PHP	*Pistachia vera*	Hulls	371, 685	Man; Xyl; GalA; Glu; Rib; GluA	-	[45]

## Data Availability

No new data were created or analyzed in this study. Data sharing is not applicable to this article.
